# Lipolysis-driven communication between adipose tissue and liver: a key axis in metabolic regulation

**DOI:** 10.1016/j.jlr.2026.101150

**Published:** 2026-09-25

**Authors:** Sicheng Zhang, Lucydalila Cedillo, Claudio J. Villanueva

**Affiliations:** Department of Integrative Biology and Physiology, University of California, Los Angeles, USA

**Keywords:** adipocytes, liver, hepatocytes, lipolysis, lipid sensing, lipid droplets, mitochondria, triglycerides

## Abstract

The adipose–liver axis is central to controlling systemic energy homeostasis, with free fatty acids (FFAs) acting as critical metabolic signals that coordinate lipid metabolism between these organs. Under physiological conditions like fasting, FFAs released via adipocyte lipolysis are taken up by the liver to support β-oxidation for ketogenesis, triglyceride synthesis, and lipoprotein production. However, in obesity and insulin resistance, chronic elevation of lipolysis exceeds hepatic lipid-handling capacity, contributing to metabolic dysfunction-associated steatotic liver disease (MASLD), lipotoxicity, and inflammation. To manage fluctuating lipid loads, hepatocytes undergo dynamic organelle remodeling—particularly within mitochondria, the endoplasmic reticulum (ER), and peroxisomes—to orchestrate lipid oxidation, storage, and detoxification. These processes are tightly regulated by transcriptional programs involving nuclear receptors (e.g., PPARα, HNF4α, and LXR) and stress-responsive signaling pathways (e.g., ATF6, XBP1, and CHOP). While these adaptive networks maintain metabolic balance under stress, their dysregulation drives hepatic dysfunction in metabolic disease. Elucidating the molecular crosstalk and regulatory mechanisms that govern this shift from adaptation to dysfunction is essential to understanding the pathogenesis of liver disease in metabolic disorders. This review highlights the molecular mechanisms underlying adipose-liver communication, the role of organelle plasticity in hepatic lipid metabolism, and the transcriptional circuits that integrate these responses.

The regulation of energy balance and metabolism relies heavily on the coordinated actions of two vital organs: the adipose tissue and the liver. Together these organs control the dynamic changes in lipid metabolism that occur on a diurnal basis, in response to dietary pressures, and changes in ambient temperature (1, 2, 3). While the adipose tissue serves as the primary organ for long-term lipid storage (Fig. 1), the liver assumes the critical roles of regulating essential metabolic pathways (glucose, lipids, and proteins), detoxification, bile production, endocrine, storage, and immune function (4). To fulfill their respective functions effectively, these organs engage in constant communication through signaling pathways and metabolite exchange. This intricate interplay can significantly influence the remodeling of lipids within the liver, both in the short- and long-term. In the short term, daily fasting and refeeding cycles can trigger adaptive changes in the liver to drive catabolic and anabolic processes, respectively (5). These adaptations support the use and storage of energy, like lipids or glycogen, while in the long term, this interplay may have implications for various common chronic diseases. This review will focus on our understanding of the interplay between adipose tissue and liver in regulating lipid dynamics. Comprehending the mechanisms behind the crosstalk between the liver and the adipose tissue is of utmost importance for gaining insights into the dynamic processes that control energy storage and utilization. Such knowledge holds the key to understanding and addressing metabolic disorders such as metabolic dysfunction-associated steatotic liver disease (MASLD).

**Fig. 1:**
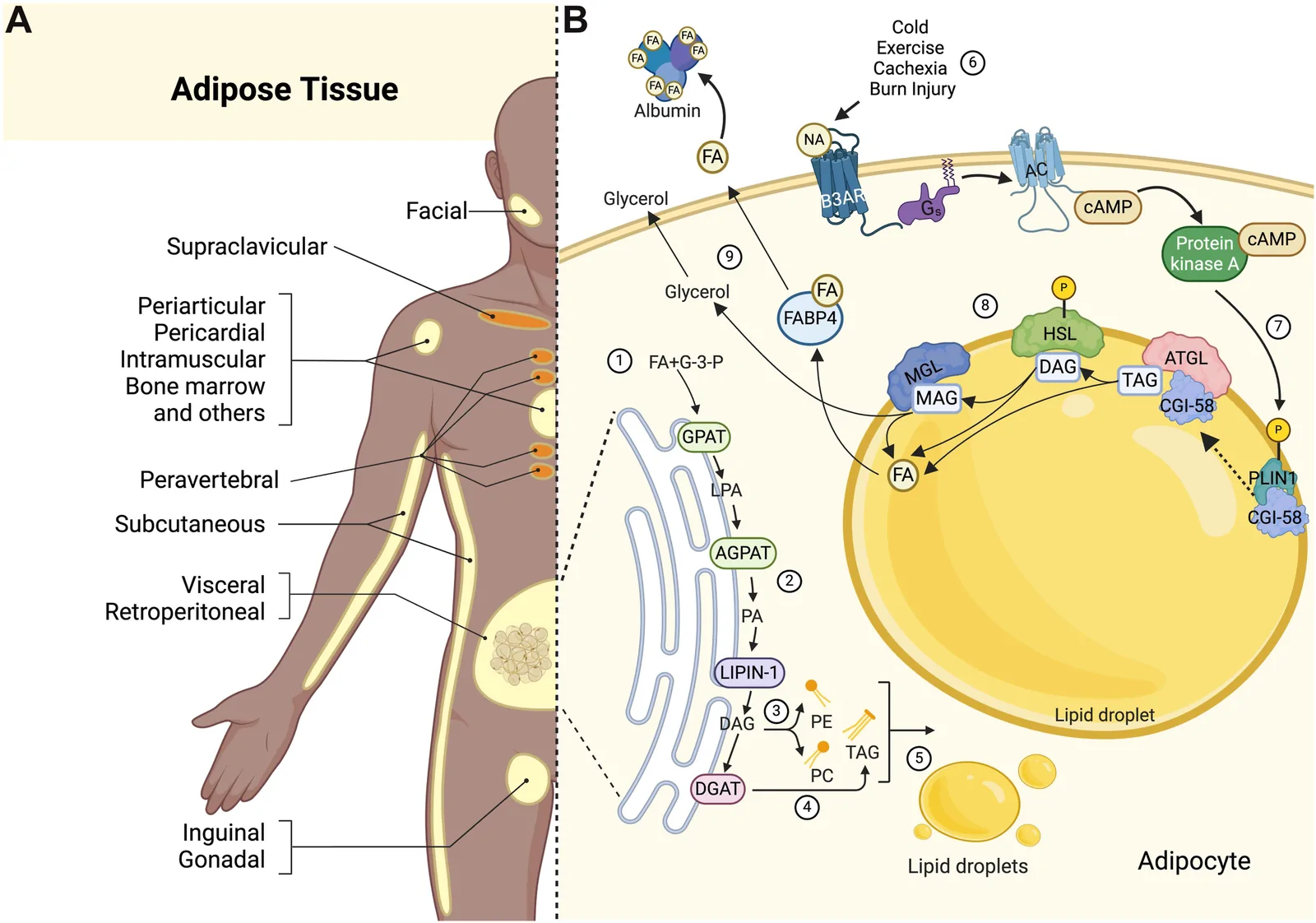
Adipose tissue depots and mechanisms of lipid mobilization. A: Adipose tissue is found in multiple locations throughout the body. White adipocytes are found in depots that include facial, subcutaneous, visceral, inguinal/gonadal, pericardial, intramuscular, bone marrow and periarticular. While thermogenic adipocytes like brown/beige are dispersed within supraclavicular, paravertebral, and retroperitoneal. B: Fatty acids are stored in adipocytes as triglycerides through glycerolipid synthesis. 1) Using glycerol-3-phosphate and acyl-CoA, GPAT generates lysophosphatidic acid, and addition of another acyl-CoA by AGPAT to generate phosphatidic acid. Upon removal of phosphate by Lipin enzymes adipocytes generate diacylglycerol (DAG), the substrate for DGAT enzymes that esterify an acyl-CoA to generate triglycerides (TG). External stimuli like cold, exercise, fasting, and burn injury increase sympathetic tone and raise hormones like nor-adrenaline (nor-epinephrine) that activate the β3-adrenergic receptor (β3-AR). These conditions increase cAMP levels and activate PKA, leading to phosphorylation of Plin1, releasing CGI-58 to activate ATGL to start the hydrolysis of triglycerides (TAG) to release diacylglycerol (DAG) and FA. Hormone-sensitive lipase (HSL) and Monoacylglycerol Lipase (MGL) then hydrolyze DAG and monoacylglycerol (MAG), respectively. Fatty acids released then adhere to cytosolic fatty acid binding protein 4 (FABP4) and are released into circulation where they adhere to albumin.

## Adipose tissue subtypes and lipid metabolism

Adipocytes are dispersed in multiple places in the body, with white adipocytes in subcutaneous and visceral adipose tissue representing the largest proportion (Fig. 1A). White adipocytes can be found in the face, periarticular, pericardial, intramuscular, and bone marrow, while thermogenic adipocytes are found in the supraclavicular, axillary, and paravertebral region. There are at least three distinct types of adipose tissue, which have emerged as a means of safeguarding against food scarcity and fluctuations in ambient temperature. These include white adipocytes, brown adipocytes, and beige adipocytes (6, 7). When subjected to cold exposure, a fascinating phenomenon occurs—thermogenic adipocytes simultaneously uptake fatty acids, glucose, and other substrates from the bloodstream to provide fuel for thermogenesis. This process aids in generating heat and aids in maintaining the body's temperature during cold conditions. Conversely, white adipose tissue primarily serves as an energy reservoir that can be used during times of caloric deficiency, storing excess energy for future use (8, 9, 10). While in beige adipocytes there is evidence of futile cycling, such as lipid cycling where TG synthesis and TG hydrolysis are occurring simultaneously, leading to a net consumption in ATP(11, 12).

## Lipogenesis and triglyceride synthesis

Triglycerides were first identified in the early 19th century. This discovery is generally credited to the French chemist Michel Eugène Chevreul, who in 1813 began studying animal fats and plant oils. He coined the term “glycéride” in 1815 to describe the fatty acid esters of glycerol—what we now call triglycerides. Chevreul’s pioneering work laid the foundation for lipid chemistry and provided a deeper understanding of the chemical nature of triglycerides (13). More than 60 years ago, Eugene Kennedy and coworkers mapped the Endoplasmic Reticulum (ER)-based pathways of glycerolipid synthesis, including the synthesis of phospholipids and triacylglycerols (14). The reactions of the Kennedy pathway were identified by isotope tracing of metabolic intermediates in lipid synthesis and isolating biochemical enzymatic activities using in vitro based approaches. The molecular basis of triglyceride synthesis came decades later with the advent of molecular biology techniques that led to the isolation of genes encoding individual enzymes involved in glycerolipid synthesis.

Triglyceride synthesis (lipogenesis) is facilitated by a distinct but complementary set of enzymes (15). This anabolic pathway involves sequential acylation reactions primarily catalyzed by glycerol-3-phosphate acyltransferase (GPAT), acylglycerol-3-phosphate acyltransferase (AGPAT), phosphatidic acid phosphatase (Lipin), and diacylglycerol acyltransferase (DGAT) (Fig. 1B). The synthesis of triglycerides begins with the acylation of glycerol-3-phosphate, a reaction catalyzed by the GPAT enzyme—the rate-limiting enzyme in the glycerol phosphate pathway. GPAT transfers a fatty acyl-CoA to the sn-1 position of glycerol-3-phosphate, forming lysophosphatidic acid (LPA), the first committed intermediate in de novo phospholipid and triglyceride biosynthesis (16). The enzymatic activity of GPAT was first described in the 1950s in mitochondrial fractions, but it was not until the late 1990s and early 2000s that distinct GPAT isoforms were identified and characterized using molecular biology techniques (17, 18, 19). To date, four GPAT isoforms have been described in mammals with varied cellular localization and tissue distribution. GPAT1 is found in the outer mitochondrial membrane with high expression in liver and adipose tissue, and genetic deletion of Gpat1 in mice results in reduced hepatic TAG accumulation and protection from diet-induced hepatic steatosis (20, 21, 22). GPAT2 localizes to the outer mitochondrial membrane in spermatocytes and mice lacking Gpat2 have impaired spermatogenesis (23). GPAT3 is found in the ER with highest expression in adipose tissue and small intestine, with Gpat3 deficiency reducing GPAT activity by approximately 80% in white adipose tissue but produces relatively modest metabolic effects under chow-fed conditions, although protection from diet-induced adiposity is observed, particularly in females (24). GPAT4 localizes to the ER with expression in adipose tissue, liver, and mammary gland, where Gpat4-deficient mice show reduced hepatic TAG content, decreased adiposity—particularly subdermal adipose tissue—and impaired mammary gland lipid synthesis (25, 26).

The second step in de novo triglyceride synthesis involves the formation of phosphatidic acid (PA) from lysophosphatidic acid (LPA), catalyzed by the enzyme 1-acylglycerol-3-phosphate O-acyltransferase (AGPAT), also known as lysophosphatidic acid acyltransferase (LPAAT). This reaction adds a fatty acyl-CoA to the sn-2 position of LPA to yield PA, a critical intermediate that channels lipid intermediates toward either triacylglycerol (TAG) or phospholipid biosynthesis. The enzymatic activity of AGPAT was first characterized in the 1970s through isotopic labeling and biochemical assays that revealed microsomal incorporation of acyl-CoAs into LPA to form PA(27). However, the molecular identification of AGPAT enzymes came decades later. In 1994 the first AGPAT cDNA was identified through complementation studies using an E. Coli strain lacking AGPAT activity and a maize cDNA library (28). This cDNA had homology to the E. Coli AGPAT gene identified by positional cloning in 1992 (29). These studies led to the identification of other AGPAT enzymes, of which there are 11 AGPAT isoforms that have been cloned (AGPAT1-AGPAT11), each with distinct tissue distribution, substrate preference, and subcellular localization (30, 31, 32). The canonical AGPAT enzymes involved in glycerolipid synthesis are integral membrane proteins localized primarily to the ER(33). AGPAT1 and AGPAT2 are the best characterized LPA acyltransferases that participate in glycerolipid synthesis. AGPAT1 has broad tissue distribution, and *Agpat1*-deficient mice exhibit reduced body weight and adiposity, hypoglycemia, reproductive abnormalities, and high preweaning mortality, although they do not develop generalized lipodystrophy (34). In contrast, AGPAT2 is highly expressed in adipose tissue and plays an important role in adipocyte TAG synthesis. Consistent with this function, *Agpat2*-deficient mice exhibit severe lipodystrophy, insulin resistance, and hepatic steatosis (35). Some isoforms, such as AGPAT3 and AGPAT4, have confirmed LPA acyltransferase activity but may also play roles in phospholipid remodeling. The broader AGPAT/acyltransferase family includes enzymes with diverse substrate specificities and functions in glycerolipid and phospholipid metabolism, and their classification and biochemical properties have been reviewed extensively elsewhere (27).

The next step in triglyceride synthesis is catalyzed by Lipin proteins, which function as phosphatidic acid phosphatases (PAPs) (36). Lipins hydrolyze phosphatidic acid (PA) to generate diacylglycerol (DAG), a key branch-point intermediate used in both triacylglycerol (TAG) and phospholipid synthesis. This reaction is essential for TAG synthesis and plays a critical role in channeling DAG into the biosynthesis of membrane phospholipids such as phosphatidylcholine (PC) and phosphatidylethanolamine (PE), depending on cellular needs. Lipins represent a family of evolutionarily conserved enzymes, with three isoforms in mammals: Lipin-1, Lipin-2, and Lipin-3(37). Lipins are soluble proteins that dynamically translocate between the cytosol, ER membrane, and nucleus (38). Lipin-1 is highly expressed in adipose tissue, skeletal muscle and testis, whereas lipin-2 is prominently expressed in liver (39, 40). Lipin-1 deficiency in fld mice causes severe lipodystrophy and fatty liver; however, hepatic PAP activity remains normal, consistent with an important contribution of lipin-2 to PAP activity in liver (39, 41). In addition to their enzymatic function, lipins can regulate transcription, with lipin-1 functioning as a transcriptional co-regulator of PGC-1α/PPARα-dependent oxidative programs in hepatocytes (42). By controlling the conversion of PA to DAG, Lipin proteins serve as gatekeepers at a metabolic branchpoint that integrates lipid storage, membrane synthesis, and metabolic regulation.

The final step in TG synthesis is catalyzed by acyl-CoA:diacylglycerol acyltransferase (DGAT) enzymes that are encoded by two distinct genes, DGAT1 and DGAT2, both of which are primarily localized to the ER(43, 44, 45). DGAT enzymes catalyze the addition of a third fatty acyl-CoA to diacylglycerol, forming triglycerides in the ER that are subsequently stored within lipid droplets. Notably, DGAT1 expression is restricted to the ER, while DGAT2 can be found associated with the ER, lipid droplets, and mitochondrial-associated membranes (46). Despite catalyzing the same reaction, DGAT1 and DGAT2 are evolutionarily unrelated (45). DGAT1 belongs to the Membrane-Bound O-Acyltransferases (MBOAT) that are related to ACAT (Acyl-CoA Acyltransferase) enzymes, while DGAT2 belongs to a distinct family (47). Mice lacking DGAT1 exhibit reduced adiposity and increased energy expenditure. This phenotype is associated with heightened insulin and leptin sensitivity, contributing to resistance against diet-induced obesity (48). In contrast, deletion of DGAT2 results in severe reductions in TG levels and leads to early postnatal death (49). The interplay between enzymes driving triglyceride breakdown and those synthesizing triglycerides is tightly regulated to maintain adipocyte lipid homeostasis.

## Transcriptional regulation of lipogenesis

Hepatic lipogenesis is tightly regulated by a network of nutrient- and hormone-responsive transcription factors that coordinate the expression of genes involved in fatty acid and triglyceride biosynthesis. Among these, sterol regulatory element-binding protein 1c (SREBP1c) is a central driver of de novo lipogenesis, activated by insulin during the fed state (50). Once cleaved and translocated to the nucleus, SREBP1c upregulates key enzymes involved in glycolysis like glucokinase (GK) and fatty acid synthesis, including fatty acid synthase (FASN), acetyl-CoA carboxylase 1 (ACC1), and stearoyl-CoA desaturase 1 (SCD1) (51, 52, 53). Working in parallel, carbohydrate-responsive element-binding protein (ChREBP) responds to glucose metabolism, particularly via xylulose-5-phosphate–mediated signaling, to induce overlapping and complementary lipogenic genes such as ACLY, ACC1, and FASN, as well as genes involved in glucose metabolism, like (GK) and Liver-Pyruvate Kinase (L-PK) (54, 55). ChREBP is stabilized by Max-like protein factor X (MLX), which acts as a heterodimer binding partner with ChREBP and inhibition of MLX has been shown to alleviate intrahepatic triglyceride accumulation in mice (56). Liver X receptor alpha (LXRα), activated by oxysterols, also promotes lipogenesis indirectly by inducing SREBP1c and other lipogenic genes, thereby linking cholesterol sensing to fatty acid synthesis (56). In pathological contexts such as hepatic steatosis, PPARγ can become aberrantly expressed in hepatocytes and drive lipid uptake and storage (57). Together, this network of transcriptional regulators integrates hormonal and nutrient cues to maintain lipid homeostasis in the liver, and its dysregulation contributes to metabolic disorders such as non-alcoholic fatty liver disease (NAFLD) and insulin resistance.

## Dynamics of triglyceride hydrolysis

The coordinated release and synthesis of fatty acids that are needed to meet the energy demands of multiple organs systems is an essential physiological role of adipocytes (58, 59, 60, 61). In the mid-19^th^ century, Claude Bernard found that pancreatic juice of mammals could efficiently degrade fat from butter. Thus, early studies to understand breakdown of fats were largely focused on pancreatic lipase (62). Indirect evidence that there is internal fat breakdown came as early as 1915 from prolonged fasting studies showing a decrease in respiratory quotient towards ∼0.7, which was interpreted as evidence of increased fat oxidation (63). This evidence suggested that stored triglycerides were being mobilized and hydrolyzed to supply energy as fatty acids. The hydrolysis of triglycerides in adipose tissue involves several key enzymes that play crucial roles in breaking down stored fat into free fatty acids and glycerol. Some of the primary enzymes involved in this process carryout sequential hydrolysis of ester bonds found in triglycerides (Fig. 1B). Adipose Triglyceride Lipase (ATGL) is the enzyme responsible for the initial step in triglyceride hydrolysis (64, 65, 66). It specifically catalyzes the conversion of triglycerides into diacylglycerol and free fatty acids. Hormone-Sensitive Lipase (HSL) further hydrolyzes diacylglycerol into monoacylglycerol and free fatty acids (67, 68, 69, 70). HSL activity is regulated by hormonal signals, such as epinephrine and insulin, which modulate its phosphorylation state and activity (71). While Monoglyceride Lipase (MGL) catalyzes the final step in the hydrolysis of monoacylglycerol into glycerol and free fatty acids (72, 73).

ATGL was first identified in 2004 by three independent research groups. It was discovered as a novel lipase involved in the initial step of TAG hydrolysis in adipocytes (64, 65, 74). The identification of ATGL filled a significant gap in the understanding of lipid metabolism, particularly the regulation of lipid stores and mobilization of fatty acids (75). The human ATGL gene is located on chromosome 11p15.5 and consists of 10 exons and is encoded by the gene patatin-like phospholipase family, characterized by a patatin-like domain 2 (Pnpla2) (74). The enzyme contains a catalytic dyad, essential for its hydrolytic function (76). ATGL activity can be regulated by phosphorylation. For instance, protein kinase A (PKA) has been shown to phosphorylate ATGL and subsequently modulating its activity (77). ATGL activity is regulated by its interaction with perilipin-1 on the surface of lipid droplets (78). Perilipin-1 phosphorylation during lipolysis facilitates hydrolysis of TAG substrates (79). Hormones like adrenaline and noradrenaline activate ATGL through the β-adrenergic receptor pathway, leading to PKA-mediated phosphorylation of perilipin-1 and subsequent activation of ATGL. Dissociation of ATGL-PLIN1 complex allows access to Comparative Gene Identification-58 (CGI-58), a co-activator of ATGL that enhances ATGL-mediated lipolysis by binding to the enzyme and increasing its catalytic efficiency (80). ATGL is hormonally regulated; when insulin levels are high, as in the fed state, PLIN1 is dephosphorylated, leading to inhibition of ATGL activity (81, 82). Additional studies interrogating the interactions of perilipins with ATGL found that Plin5 interacts with ATGL displacing its coactivator ABHD5, reducing ATGL activity (83).

Hormone-sensitive lipase (HSL) hydrolyses DAG to release an acyl chain and mono-acylglycerol (71, 84, 85). Early biochemical studies showed that HSL is activated by catecholamines, through the cAMP-dependent protein kinase A (PKA) pathway, which phosphorylates HSL and enhances its lipolytic activity (86). Insulin, conversely, inhibits HSL activity by decreasing cAMP levels and preventing the enzyme’s phosphorylation (86). HSL has 10-fold greater substrate specificity with DAG when compared to TAG((87)). HSL plays a pivotal role in adipocytes but is also expressed in tissues like ovary, adrenal, testis, intestines, heart, and lung, where it is involved in the hydrolysis of other lipid substrates such as cholesterol esters (88, 89). HSL contains an essential catalytic triad consisting of serine, aspartate, and histidine residues at the active site, which are responsible for its lipase activity (70, 90). HSL contains key phosphorylation sites, such as Ser563, Ser659, and Ser660, that are crucial for the enzyme’s activation by PKA((91, 92)). Additionally, the discovery of the interaction between HSL and the lipid droplet-associated protein perilipin helped elucidate the mechanism by which HSL gains access to stored lipids in adipocytes. When perilipin is phosphorylated by PKA, it undergoes conformational changes, allowing HSL to translocate to the lipid droplet and initiate lipolysis (93).

Biochemically, HSL belongs to the esterase/lipase family and exhibits broad substrate specificity, hydrolyzing not only triglycerides but also diacylglycerols, monoacylglycerols, and cholesterol esters. However, loss-of-function studies targeting HSL show a significant rise in DAG, suggesting DAG as a preferred substrate (94). Its catalytic activity is governed by a multi-domain structure, with an N-terminal domain containing the active site and a C-terminal domain involved in substrate binding and interaction with lipid droplets. HSL is under dynamic regulation and its interaction with intracellular lipids ensures precise control over lipid mobilization. Dysfunction of HSL is implicated in various metabolic disorders, including obesity, insulin resistance, and type 2 diabetes, as impaired HSL activity can lead to excessive lipid accumulation in both adipose and non-adipose tissues.

MGL specifically catalyzes the hydrolysis of monoglycerides into free fatty acids and glycerol. It is primarily responsible for breaking down 2-arachidonoylglycerol (2-AG), a bioactive lipid that serves as a key endocannabinoid, making MGL crucial not only in lipid metabolism but also in modulating endocannabinoid signaling pathways (95). MGL is expressed in various tissues, including the brain, liver, and adipose tissue, and plays an essential role in regulating the levels of monoglycerides that result from the breakdown of triglycerides and diacylglycerols during lipolysis (96). Mice lacking MGL show significant body weight reduction when challenged with a high-fat diet, an outcome that likely reflects enhanced futile cycling that promotes energy utilization (97, 98). In the central nervous system, MGL plays a pivotal role in maintaining the balance of 2-AG, which influences neurotransmitter release and various physiological processes, such as appetite, pain sensation, and inflammation (99).

From a biochemical perspective, MGL belongs to the serine hydrolase family, similar to HSL, and contains a catalytic triad composed of serine, histidine, and aspartate residues. The enzyme exhibits substrate specificity for monoglycerides, with a preference for 2-arachidonoylglycerol, and operates at the terminal stage of the lipolytic pathway to ensure the complete hydrolysis of lipids. The cloning of MGL was first achieved in 1997, which facilitated the study of its structure and function (73, 100). The cloned enzyme revealed the presence of conserved serine hydrolase domains, and subsequent studies demonstrated the enzyme’s ability to degrade monoacylglycerols, thus playing a vital role in terminating the signaling of lipid-derived molecules like 2-AG(101). The identification of MGL’s role in the degradation of 2-AG positioned it as a target of interest in research on neurodegenerative diseases, pain management, and metabolic disorders.

Regulation of MGL activity is crucial for maintaining proper lipid and endocannabinoid homeostasis. Dysregulation of MGL can lead to altered levels of 2-AG, which has been implicated in a variety of conditions, including obesity, inflammation, and neurodegeneration. Inhibition of MGL has emerged as a therapeutic strategy to prolong 2-AG signaling, particularly in the context of pain relief and neuroprotection (102). However, maintaining balance is key, as excessive inhibition may lead to unwanted side effects, such as impaired lipid metabolism or endocannabinoid system dysfunction. Consequently, MGL remains a key focus in therapeutic research aimed at modulating lipid signaling and energy metabolism, offering potential treatments for a range of diseases from metabolic syndromes to central nervous system disorders.

## Hormone-dependent regulation of lipolysis

The release of non-esterified fatty acids (NEFAs) from adipose tissue is under the dynamic regulation of circulating hormones like insulin and catecholamines like epinephrine and norepinephrine. Epinephrine is released by the adrenal medulla and sympathetic nerve endings that signal through adrenergic receptors (ARs) (103). ARs are G-protein-coupled receptors on the plasma membrane that simulate lipolysis in white adipose tissue (WAT) and thermogenesis in brown adipose tissue (BAT). Adipocytes express all three beta-adrenergic receptors (β1,β2,β3-ARs) with β3 being the most abundant (104, 105, 106). Upon activation, the Gs from G-protein interacts with adenylyl cyclase (AC) to stimulate cAMP (cyclic AMP) production and subsequently activates cAMP-dependent protein kinase, protein kinase A (PKA), which is a critical regulator of lipolysis. PKA subsequently phosphorylates perilipin 1, leading to the release of ABHD5, which then activates ATGL, significantly enhancing lipolysis (107). βAR activation is critical in promoting energy expenditure and mobilization in mice, as loss of all β-ARs in adipose tissue results in obesity when mice are challenged with a high-fat diet (108).

The suppression of NEFA release from adipose tissue is primarily mediated by insulin, a key anabolic hormone secreted by pancreatic β-cells in response to nutrient intake. Insulin exerts potent anti-lipolytic effects by activating the insulin receptor (IR), a receptor tyrosine kinase found in the plasma membrane of adipocytes (109). IR engagement triggers phosphorylation cascades via the insulin receptor substrates (IRS), which activate phosphatidylinositol 3-kinase (PI3K) and downstream Akt (protein kinase B). Insulin promotes the accelerated degradation of cAMP by enhancing the activity of the cAMP-degrading enzyme phosphodiesterase 3B (PDE3B), resulting in decreased activation of PKA(110). In parallel, insulin promotes dephosphorylation of HSL and perilipin 1 through the activation of phosphatases such as protein phosphatase 1 (PP1), further dampening lipolytic activity (92, 111, 112). Moreover, insulin increases lipid storage by upregulating lipogenic genes and promoting triglyceride synthesis (113). In insulin-resistant states, such as obesity and type 2 diabetes, these inhibitory effects on lipolysis are blunted, leading to uncontrolled NEFA release and ectopic lipid deposition in insulin-sensitive tissues including liver and muscle (114).

## Adipose tissue lipolysis through cold, exercise, and fasting

Cold exposure triggers a rise in energy expenditure and activates a tightly coordinated signaling network between adipose tissue and the liver, establishing a well-characterized model of interorgan metabolic communication. The cold is sensed through sensory neurons that are ultimately transmitted through sympathetic neural pathways, leading to the release catecholamines such as epinephrine. This, in turn, activates β-adrenergic receptors on adipocytes, initiating a cAMP-PKA signaling cascade that drives lipolysis and non-shivering thermogenesis (115, 116). Concurrently, cold exposure stimulates the expression of genes involved in thermogenesis, such as uncoupling protein 1 (UCP1) and PPARγ coactivator 1-alpha (PGC1α), through the action of transcriptional regulators like transcription factor 2 (ATF2). The browning of WAT is marked by increased mitochondrial biogenesis and the expression of thermogenic pathways, promoting energy utilization. Studies in ATGL-deficient mice have shown a significant impairment in lipid mobilization that results in cold sensitivity due to the inability to supply sufficient FFAs for thermogenesis (117). In parallel, the liver adapts to cold stress by increasing its production of acyl-carnitines, critical intermediates for mitochondrial fatty acid oxidation. Hepatic transcription factors such as hepatocyte nuclear factor 4-alpha (HNF4α) and peroxisome proliferator-activated receptor alpha (PPARα) orchestrate the upregulation of enzymes involved in fatty acid β-oxidation and ketogenesis, ensuring a continuous supply of energy substrates during prolonged cold exposure (118, 119). Deficiencies in hepatic HNF4α in mouse models have been shown to exacerbate cold sensitivity, underscoring the liver's pivotal role in systemic energy balance during thermal stress. This intricate interplay between adipose tissue and liver highlights the coordinated regulation of metabolic pathways essential for survival in cold environments (119).

Exercise represents a significant form of acute energy expenditure that mimics certain aspects of cold exposure by enhancing lipolysis in adipose tissue to provide FFAs for muscle, while subsequently stimulating the expression of thermogenic and metabolic genes in subcutaneous WAT, including genes like UCP1 and PGC1α(120). Exercise exerts beneficial effects on MASLD by reducing hepatic fat accumulation and driving energy utilization. These effects are mediated, in part, by the suppression of de novo lipogenesis pathways and attenuation of inflammatory signaling (121). Through the mobilization of fats in adipose tissue, exercise activates receptors such as PPARα and HNF4α, thereby augmenting lipid metabolism. Moreover, there is a substantial elevation in the processes of β-oxidation and the tricarboxylic acid (TCA) cycle within skeletal muscle. As a collective consequence, energy expenditure by the muscle and liver can effectively counteract lipid buildup within these organs (120, 122).

Fasting has emerged as an intervention aimed at extending both lifespan and overall health by reducing oxidative and metabolic stresses, cardiovascular risks, obesity, and MASLD. Fundamentally, fasting causes a shift from glucose metabolism to the utilization of circulating FFAs and the production of ketones, concurrently dampening the activation of mechanistic target of rapamycin (mTOR)-mediated nutrient signaling pathways (123). Fasting drives the release of FFAs into circulation, leading to influx of lipids into various organs, including the liver, heart and muscle. In the fasting state, the predominant energy source transitions to fats and ketones (124). Furthermore, fasting escalates the process of autophagy within the liver, facilitating the breakdown of proteins, glycogen, lipids, as well as damaged organelles like mitochondria and portions of the ER(125). However, recent studies have proposed that both fasting and subsequent refeeding disproportionately amplify the expression of genes linked to lipogenesis in adipose tissue and the liver (126).

## Adipose tissue lipolysis provides signals and substrate for hepatic lipid remodeling

Fatty acids (FAs) circulating in the bloodstream are predominantly bound to serum albumin and delivered to the liver, especially during fasting or increased lipolysis in adipose tissue. Hepatocytes take up NEFAs through a combination of passive diffusion and facilitated transport mechanisms involving proteins such as CD36, fatty acid transport proteins (FATPs)—like FATP5—and plasma membrane fatty acid-binding protein (FABPpm) (127, 128, 129). Once inside the hepatocyte, fatty acids are sequestered by liver fatty acid-binding protein (L-FABP), which solubilizes these hydrophobic molecules and directs them to membranes of different intracellular compartments (Fig. 2). L-FABP is found in the cytosol and has the capacity to carry two fatty acids simultaneously (130). The metabolic fate of these fatty acids is determined by nutrient status and hormonal cues: they can undergo β-oxidation in mitochondria to generate ATP, be re-esterified into triglycerides (TAG) for storage in lipid droplets or packaging into very-low-density lipoproteins (VLDL) for export, or be incorporated into phospholipids for membrane synthesis (50). A rise of intracellular fatty acids occurs in parallel to changes in FA derivatives such as branched-chain FAs, phospholipids, acyl-carnitines, and branched fatty acid (FA) esters of hydroxy FAs (HFAs; FAHFAs) (6, 115, 131). There is evidence to suggest that release of fatty acids from adipocytes is facilitated by cytosolic fatty acid–binding proteins like FABP4 that are released into circulation (132, 133). The composition of FFAs released during adipocyte lipolysis includes long-chain fatty acids like palmitate (C16:0), oleate (C18:1), and linoleate (C18:2), which make up the majority of fatty acid species found in the TG pool of adipose tissue (134). In contrast, very long chain fatty acids (VLCFAs, over 20 carbons) and other polyunsaturated fatty acids rise in response to a lipolytic stimulus, but to a lesser degree (134). Conditions like cold exposure and fasting for example, can lead to significant remodeling of lipids in the liver and enhanced ketogenesis (119). During lipolysis, FFAs in circulation serve several purposes, including acting as substrates for CPT1 to generate acyl-carnitines and ketones, or as substrates for triglyceride (TG) synthesis, and as signals to activate transcription factors like PPARs and HNF4a (115, 119). TGs generated in the liver can be subsequently exported out of cells by the very low-density lipoprotein (VLDL) pathway to supply energy to other organs involved in lipid consumption, like BAT, muscle, and heart (135).

**Fig. 2:**
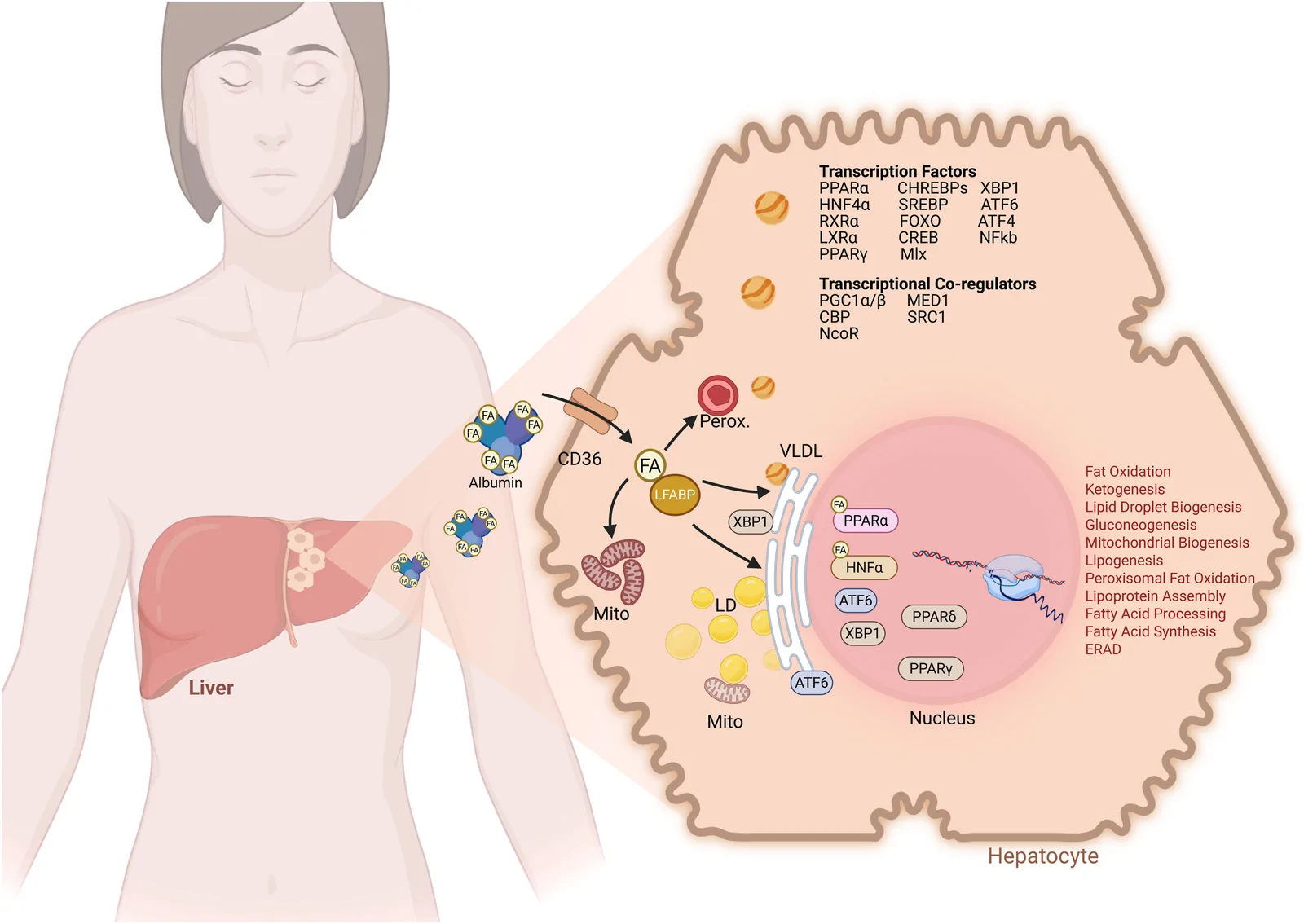
Fatty acid signaling and metabolism in the liver. Fatty acids in the blood circulate bound to albumin and are transported into hepatocytes through CD36. Once in the cell, fatty acids are bound by liver-fatty acid binding protein (LFABP) and are then oxidized by mitochondria, peroxisomes or esterified in the Endoplasmic Reticulum (ER) to generate triglycerides and stored in lipid droplets (LD). Triglycerides can be secreted as VLDL and transported to other cell types. Fatty acids can also act as signals to directly regulate PPARα and HNF4α, while the ER stress response drives the activation of ATF6 and XBP1 to promote remodeling of the ER. Thus, lipid metabolism in the liver is coordinated by multiple transcription factors and transcriptional co-regulators.

## Shifting organelle dynamics in response to adipose tissue lipolysis

Fatty acids are not merely fuel or building blocks; they act as signals that drive the dynamic interplay among cellular organelles, including mitochondria, ER, lipid droplets, lysosomes, and peroxisomes. In healthy physiology, this interplay allows cells to remodel organelles and adjust their interactions to accommodate metabolic shifts – for instance, proliferating peroxisomes and mitochondria to burn fat during fasting, or expanding ER and LD capacity to store fat after a high-fat meal (Fig. 2). These organelles continuously communicate via contact sites and vesicular pathways to ensure fatty acids are efficiently channeled to their proper fates. By coordinating oxidation, storage, and recycling of FAs, the organelle network protects the cell from lipotoxic injury while meeting energy demands. Proper coordination of organelle dynamics ensures fatty acids are oxidized for energy or stored safely whereas imbalances or overload can trigger organelle stress, remodeling, and contribute to metabolic diseases like MASLD. Below, we summarize the roles of FAs in regulating the function and interactions of each organelle, the mechanisms driving organelle remodeling and crosstalk.

## ER–lipid droplet

The ER is the major site of TG biosynthesis and is the starting point for lipid droplet synthesis (Fig. 2). The ER membrane-bound enzymes, such as DGAT, are responsible for synthesizing TGs from fatty acids and glycerol-3-phosphate (136). Some of these fatty acids that are not immediately used for energy can be stored in the liver in the form of TGs within cytosolic lipid droplets (LD) (137). The newly synthesized TGs are then incorporated into the growing LD within the phospholipid membrane of the ER, leading to LD formation and expansion. Since LDs originate from the ER, they often remain tethered to the ER membrane. ER–LD contacts are essential for LD growth—the ER supplies newly synthesized lipids to expanding LDs and may even share proteins (138). These contacts also allow exchange of fatty acids: for instance, when LDs shrink during lipolysis, fatty acids may transfer back to the ER for reesterification if not immediately needed for energy. ER–LD junctions are thus dynamic; fatty acid availability can increase the number of ER–LD contacts to promote efficient lipid storage. Some evidence suggests the ER stays attached to LDs via membrane bridges, and ER enzymes like DGAT2 can reside on LD monolayers to locally regenerate TAG from incoming FAs (139). This coupling ensures a rapid buffering of fatty acids into LDs, protecting other organelles from FA overload (140).

## Mitochondria–lipid droplet

Physical contacts between mitochondria and LDs have been observed in many cell types and are especially prominent in tissues with active fat metabolism (liver, muscle, and brown fat). At these contact sites, mitochondria can directly access fatty acids released by LD-localized lipases. During starvation or high FA oxidation states, cells increase the transfer of FAs from LDs to mitochondria—possibly via tethering complexes like the recently discovered VPS13D–TSG101 complex that facilitates FA flux at LD–mitochondria interfaces (141). Such contacts may be regulated by LD coat proteins (e.g., perilipins) that can bind mitochondrial proteins to dock the organelles. Interestingly, tethering of mitochondria to LDs can have context-dependent effects: in some cases, it promotes rapid FA oxidation, while in others (e.g., in heart muscle with high PLIN5) it may sequester mitochondria at LDs and dampen oxidation in favor of storage (142). Thus, mitochondrial contact with lipid droplets may provide a mechanism to drive lipid synthesis using mitochondrial-associated enzymes like GPAT and AGPAT. Regardless, mitochondrial access to LD-derived FAs is a vital communication axis, and disruption of this coupling could result in FAs becoming trapped in droplets (contributing to steatosis) or, conversely, in mitochondrial overload with FAs (causing lipotoxic stress). Emerging evidence suggests hepatocytes form unique mitochondria-LD contact structures when under metabolic stress (ethanol exposure, starvation), hinting at inducible tether adaptations to channel FAs efficiently.

## Lipid Droplet–Peroxisome

In addition to mitochondria, LDs establish functional contacts with peroxisomes, creating a direct route for lipid trafficking between these organelles. Peroxisomes preferentially oxidize very-long-chain fatty acids (VLCFAs) and other lipid species that are inefficiently processed by mitochondria, highlighting the complementary roles of these organelles in lipid catabolism. In yeast, this interaction is highly specialized, with peroxisomes extending membrane projections known as *pexopodia* into LDs to directly extract lipids for β-oxidation (143, 144). In mammals, LD–peroxisome contacts are stabilized by protein complexes that include tethering factors such as Spastin, thereby promoting efficient substrate channeling from LDs to peroxisomes for lipid catabolism (145). This interplay is particularly important during lipid excess, when peroxisomes associated with LDs can rapidly initiate β-oxidation of very-long-chain fatty acids (VLCFAs), generating shorter acyl chains that are subsequently oxidized by mitochondria or further metabolized within peroxisomes. Conversely, reduced LD–peroxisome contacts, which have been reported under conditions of severe fatty acid overload when lysosome-dependent lipid degradation predominates, may limit efficient substrate transfer and contribute to lipid accumulation and larger lipid droplets (146). While loss of peroxisomes or impaired peroxisomal function leads to lipid accumulation within LDs, further underscoring the importance of LD–peroxisome crosstalk in maintaining cellular lipid homeostasis (147).

## Lysosome–lipid droplet

Lysosomes are critical regulators of lipid metabolism, with notable contacts with LDs that directly engulf and degrade them (lipophagy) (148). These contacts are mediated by specialized tethering factors and allow the lysosomal membrane to invaginate and sequester LD content. Such direct LD uptake has been shown in hepatocytes as a mechanism to clear LDs during nutrient deprivation. Additionally, completed autophagosomes containing LDs must fuse with lysosomes, a step that functionally links these organelles. Fatty acids may also be released from lysosomes and transferred back to LDs or to mitochondria/peroxisomes. In fact, recent research suggests cells can even expel excess fatty acids via lysosome exocytosis after lipophagy, to avoid intracellular lipotoxicity(149). Thus, lysosomes communicate with LDs and other organelles in the FA network not only by degrading cargo but by coordinating the fate of the liberated fatty acids. Lysosomes are cellular organelles in hepatocytes that are involved in the degradation and recycling of cellular waste and foreign substances. They contain a variety of hydrolases that facilitate processing of large macromolecules, including lipids (150, 151). This communication helps regulate the level of FFAs in the liver and maintain energy balance.

## Adipose-to-liver signaling drives transcriptional adaptation

The liver responds to fatty acids through transcriptional mechanisms that coordinate organelle dynamics, storage, and utilization of fatty acids. This regulation is mediated by transcription factors, transcriptional co-regulators, and histone modifications. Among these, nuclear receptors are critical regulators of lipid metabolism that sense lipids or their derivatives through their respective ligand binding domains. Key nuclear receptors involved in this process include PPARs (α, β, γ1, and γ2), HNF4(α/γ), and retinoid X-receptor (RXR) (152, 153).

Among these transcription factors, PPARα plays a central role in lipid sensing. Structural and biochemical studies, including X-ray crystallography and coactivator interaction assays, have demonstrated that PPARα binds fibrates, eicosanoids, and synthetic agonists with high affinity (EC_50_ ≈ 50 nM–50 μM). In contrast, long-chain fatty acids such as palmitate, oleate, and stearate bind PPARα with lower affinity (EC_50_ ≈ 500–1,000 μM). Notably, these concentrations fall within the physiological range achieved during acute cold exposure in mice, where circulating fatty acid levels can reach ∼1,000–1,500 μM. (115, 154, 155). Notably, products of de novo fatty acid synthesis like palmitate are endogenous ligands in the liver and are necessary to activate PPARα(156). Hepatic PPARα is critical in driving the transcriptional adaptations to fasting and cold exposure, orchestrating transcriptional programs that promote fatty acid oxidation, ketogenesis, and glucose homeostasis (157). Conditional deletion of hepatic PPARα leads to increased lipid accumulation, impaired ketogenesis, and oxidative stress in liver cells (157). PPARα also regulates a variety of genes involved in lipid metabolism, including those responsible for fatty acid uptake and transport, as well as genes involved in lipoprotein metabolism (119). For example, PPARα controls the regulation of the fatty acid translocase CD36 and the fatty acid transport protein (FATP), which facilitates fatty acid uptake. PPARα also increases the expression of acyl-CoA oxidase (ACOX1), carnitine palmitoyltransferase (CPT1a/b, CPT2), and other enzymes involved in β-oxidation (158, 159, 160, 161, 162).

*Hepatocyte nuclear factor 4* (HNF4)α and HNF4γ are additional critical regulators of lipid and carbohydrate metabolism in the liver. Similar to PPARα, HNF4 is a nuclear receptor with a lipid-binding domain. There is a traditional view that HNF4’s ligand-binding domain contains a tightly bound fatty acid that is not exchangeable (163). More recently, however, there is evidence that HNF4α can undergo ligand exchange, leading to enhanced receptor activity in response to linoleate (164). HNF4α is involved in the regulation of many genes involved in various metabolic processes, including glucose and lipid metabolism, bile acid synthesis, thermogenesis, and drug metabolism (115, 165). HNF4α promotes the expression of genes involved in fatty acid oxidation, such as carnitine palmitoyltransferase I (CPT-1) and ACOX1, which catalyze the transport of fatty acids into the mitochondria and their subsequent oxidation (115). HNF4α also controls lipoprotein metabolism, including apolipoprotein A-IV (APOA4), which is involved in the transport of lipids in the bloodstream (115). Dysregulation of HNF4α activity can lead to various metabolic disorders, including dyslipidemia, insulin resistance, and hepatic steatosis. HNF4α is, therefore, a potential target for the treatment of metabolic disorders associated with lipid metabolism dysfunction.

The ER plays a crucial role in protein folding, lipid metabolism, and calcium homeostasis. However, when cells experience metabolic stress—such as an excessive influx of free fatty acids (FFAs)—the ER's protein-folding capacity can become overwhelmed, triggering ER stress. In response, cells activate the unfolded protein response (UPR), a defense mechanism that reduces ER protein load, enhances protein-folding capacity, and promotes degradation of misfolded proteins (166). While the UPR is initially protective, chronic ER stress due to sustained lipid overload is thought to contribute to metabolic dysfunction, inflammation, and cell death, which are key features of MASLD, insulin resistance, and obesity-related disorders (167). The UPR is mediated by three major ER-resident transmembrane sensors: inositol-requiring enzyme 1 (IRE1/ERN1), activating transcription factor 6 (ATF6), and protein kinase RNA-like ER kinase (PERK) (166, 168). These sensors detect ER stress signals and initiate distinct yet interconnected signaling pathways: The IRE1 (ERN1)–XBP1 Pathway generates active XBP1s, a potent transcription factor that upregulates genes involved in ER expansion, lipid biosynthesis, and protein quality control to increase lipid loads. However, chronic activation of XBP1s can contribute to hepatic lipid accumulation and steatosis. During ER stress, ATF6 translocates from the ER to the Golgi apparatus, where it undergoes proteolytic cleavage, releasing its active cytosolic fragment (ATF6f) (169). This fragment migrates to the nucleus and activates genes encoding ER chaperones (e.g., BiP/GRP78, GRP94) and lipid metabolism regulators. Thus, ATF6 is essential for restoring ER homeostasis. In contrast, the PERK–ATF4 Pathway phosphorylates eukaryotic initiation factor 2α (eIF2α), which reduces global protein synthesis to alleviate ER burden (170).

## Transcriptional coregulators that integrate metabolic response

Transcriptional coregulators, including coactivators and corepressors, are central to the dynamic regulation of gene expression in response to metabolic cues. Although they do not directly bind DNA, these proteins function as molecular scaffolds and enzymatic adaptors that interact with sequence-specific transcription factors to fine-tune transcriptional programs. In the context of metabolic homeostasis, coregulators serve as critical nodes that integrate intracellular nutrient, hormonal, and redox signals to coordinate gene expression with physiological demand. Many metabolic coregulators exert their function by recruiting chromatin-modifying enzymes, thereby controlling the accessibility of metabolic gene loci. For example, coactivators like PGC-1α and p300/CBP recruit histone acetyltransferases (HATs) to activate genes involved in mitochondrial biogenesis and oxidative metabolism, while corepressors such as NCoR and SMRT recruit HDACs to silence lipogenic or inflammatory gene programs. These interactions are highly responsive to nutrient and hormonal cues, enabling flexible chromatin remodeling in metabolic tissues such as liver and adipose tissue (171, 172).

PGC-1α (*PPARGC1A*) was first identified as a cold-inducible coactivator of PPARγ in brown adipose tissue, where it drives adaptive thermogenesis (173). PGC1α is a transcriptional coactivator that interacts with multiple nuclear receptors and transcription factors, including PPARs, HNF4α, FOXO1, and ERRa (174). PGC1α serves as a central regulator of mitochondrial biogenesis, oxidative metabolism, and gluconeogenesis (172). In response to energy demands, such as fasting, cold exposure, and exercise, PGC1α enhances fatty acid oxidation and thermogenesis by coactivating PPARα and estrogen-related receptor alpha (ERRα) in the liver and muscle. In hepatocytes, PGC1α also upregulates two key enzymes in gluconeogenesis, glucose-6-phosphatase (G6Pase) and phosphoenolpyruvate carboxykinase (PEPCK), thereby increasing hepatic glucose output during fasting. Additionally, PGC1α interacts with nuclear factor erythroid 2-related factors (NRF)half to stimulate mitochondrial DNA transcription, ensuring adequate ATP production under energy stress (175).

The first nuclear receptor coactivator to be identified was steroid receptor coactivator 1 (SRC-1/NCOA1), a founding member of the p160 family of nuclear receptor coactivators, enhancing ligand-dependent transcriptional activation of nuclear receptors like estrogen receptor, PPAR, and thyroid hormone receptors (TRs) (176). SRC-1 binds through LXXLL motifs that recognize the activation function-2 (AF-2) domain of nuclear receptors acting as a scaffold for multiprotein complexes that interact with enhancers. SRC-1 plays a key role in lipid metabolism, glucose homeostasis, and thermogenic regulation (177, 178). In the liver, SRC-1 regulates lipogenic and gluconeogenic genes, impacting overall metabolic balance (177). Additionally, SRC-1 has been implicated in sex hormone-dependent metabolic regulation, with estrogen receptor-SRC1 interactions influencing lipid storage and insulin sensitivity. Loss of SRC-1 function has been associated with obesity and metabolic dysregulation due to impaired energy expenditure (179).

CREB-binding protein (CBP) is a transcriptional coactivator that interacts with CREB (cAMP response element-binding protein) and various nuclear receptors via the AF-2 domain through LXXLL motifs, enhancing ligand-dependent activation (180). CBP belongs to a family of histone acetyltransferases (HATs) that acetylate lysine residues on histones (e.g., H3K27, H3K18), promoting chromatin relaxation and transcriptional activation (181). CBP plays a vital role in cAMP-mediated gene expression, which is essential for promoting hepatic gluconeogenesis. Under fasting conditions, CBP enhances PGC1α-mediated activation of gluconeogenic genes, thereby increasing glucose production (182). In hepatocytes, CBP has been implicated in the regulation of key lipogenic programs downstream of LXR and SREBP1c. LXR activation induces transcription of lipogenic genes such as *Srebp1c*, *Fasn*, and *Scd1*, and CBP contributes to this process by cooperating with LXR pathways and by acetylating and stabilizing SREBP1c, thereby enhancing its transcriptional activity (183). In the fed state, CBP thus promotes de novo lipogenesis through integration with LXR–SREBP1c–dependent circuits (184). In the fasted state, CBP can also participate in fatty acid oxidation programs through interactions with PGC-1α and PPARα (182). Thus, CBP’s role in hepatic lipid metabolism is context-dependent: promoting lipid synthesis under nutrient abundance, while supporting oxidative programs during fasting.

Mediator complex subunit 1 (MED1) is a crucial component of the Mediator complex, functioning as a molecular interface between gene-specific transcription factors and RNA polymerase II(185). The mediator complex is a multi-subunit complex conserved from yeast to humans and serves as a dynamic integrator of transcriptional regulation (186). MED1 links nuclear receptors to RNA polymerase II, including nuclear receptors like PPARs, LXRs, and FXRs, thereby regulating genes involved in lipid metabolism and glucose homeostasis. In hepatocytes, MED1 enhances PPARα-mediated fatty acid oxidation, reducing hepatic lipid accumulation. Additionally, MED1 is essential for cholesterol homeostasis, as it modulates LXR-induced expression of ATP-binding cassette (ABC) transporters, which facilitate cholesterol efflux. Loss of MED1 function leads to hepatic steatosis and dyslipidemia, an outcome driven by reduced fatty acid oxidation, and reduced autophagy and lipophagy (187).

## Metabolic dysfunction-associated steatotic liver disease and obesity

MASLD is the most prevalent cause of chronic liver disease globally, affecting up to 38% of the adult population and up to 90% of obese and 50% of diabetic patients (188, 189, 190). It is characterized by hepatic steatosis along with at least one cardiometabolic risk factor, which includes conditions like obesity, diabetes, dyslipidemia, and hypertension. MASLD is a major public health concern due to rising obesity rates, with estimates suggesting that 34% of the U.S. population and 25.2% globally are affected by fatty liver (191, 192, 193). The link between MASLD and cardiovascular disease (CVD) is particularly concerning, as MASLD is often accompanied by systemic metabolic dysregulation that predisposes individuals to atherogenesis and endothelial dysfunction. In patients with MASLD, elevated levels of free fatty acids (FFAs) and de novo fatty acid synthesis in the liver have been linked with systemic insulin resistance, a contributor to the development of CVD (191,194). When adipose tissue becomes resistant to insulin, it leads to greater release of FFAs into circulation, which can accumulate in the liver, promoting triglyceride deposition in the liver (195). Simultaneously, the liver's ability to synthesize new fatty acids from non-lipid sources like carbohydrates is also increased, further contributing to the triglyceride buildup in the liver. The hyperinsulinemia that occurs with insulin resistance promotes fatty acid synthesis through activation of the SREBP1c pathway, driving endogenous lipid synthesis (196). Furthermore, insulin resistance, independently of body weight, promotes cholesterol synthesis, an outcome contributing to CVD risk (197). Studies show that individuals with MASLD have a heightened risk of cardiovascular complications, which are a leading cause of mortality in these patients, often surpassing liver-related outcomes. In obesity, the role of adipose tissue as a fat reservoir becomes compromised, leading to lipid spillover into the liver and further metabolic stress (198). This process can worsen hepatic steatosis and accelerates the progression to metabolic-dysfunction associated steatohepatitis (MASH), cirrhosis, and liver failure. Individuals with fatty liver also show a higher risk of hepatocellular carcinoma, the most common form of liver cancer that accounts for about 90% of cases (199). Given that 90% of MASLD is associated with obesity (200), addressing this metabolic dysfunction and its impact on cardiovascular health is critical in preventing both liver and heart disease progression. Understanding the interplay between adipose tissue dysfunction, liver disease, and cardiovascular risk is essential for devising effective therapeutic interventions to mitigate the systemic complications of MASLD.

## Summary: Adipose-liver crosstalk in MASLD and metabolic integration

A central mechanism linking adipose tissue dysfunction to hepatic lipid accumulation is adipose tissue lipolysis, the breakdown of triglycerides into FFAs and glycerol, which are released into circulation and taken up by the liver. While physiological lipolysis provides essential energy substrates, excessive adipose tissue lipolysis in obesity results in FFA overload, overwhelming hepatic lipid-handling capacity and contributing to lipotoxicity, oxidative stress, and mitochondrial dysfunction in hepatocytes. To adapt to fluctuating FFA availability, hepatocytes undergo organelle remodeling, particularly within the mitochondria, ER, and peroxisomes. Increased hepatic FFA uptake necessitates enhanced mitochondrial β-oxidation, yet an imbalance between lipid oxidation and storage can lead to ER stress, peroxisomal overload, and increased lipid droplet formation. This adaptive response is transcriptionally regulated by nuclear receptors and metabolic transcription factors, including PPARα, HNF4α, and ATF6, which coordinate lipid oxidation, storage, and export to maintain homeostasis. However, under chronic metabolic stress, these adaptive mechanisms fail, exacerbating hepatic steatosis, insulin resistance, and inflammation. Understanding the transcriptional regulation and organelle adaptations that integrate adipose-liver metabolism is crucial for developing targeted therapies for MASLD and related metabolic disorders. Therapeutic strategies aimed at modulating nuclear receptor activity (e.g., PPARα agonists), reducing ER stress, or enhancing mitochondrial function hold promise for mitigating hepatic lipid overload and restoring metabolic homeostasis.

### Data availability

All data presented are contained within the manuscript.

### Funding and additional information

Funding was provided by the NIH under grants NIH U24DK132746, NIH K12GM106996, NIH R01GM154435, and NIH T32DK138892.

#### Conflict of interest

The authors declare that they have no conflicts of interest with the contents of this article.

#### Author contributions

C. J. V., L. C., and S. Z. writing–review & editing, C. J. V. and S. Z.writing–original draft, C. J. V. supervision, C. J. V. resources.
